# AI chatbots show promise but limitations on UK medical exam questions: a comparative performance study

**DOI:** 10.1038/s41598-024-68996-2

**Published:** 2024-08-14

**Authors:** Mohammed Ahmed Sadeq, Reem Mohamed Farouk Ghorab, Mohamed Hady Ashry, Ahmed Mohamed Abozaid, Haneen A. Banihani, Moustafa Salem, Mohammed Tawfiq Abu Aisheh, Saad Abuzahra, Marina Ramzy Mourid, Mohamad Monif Assker, Mohammed Ayyad, Mostafa Hossam El Din Moawad

**Affiliations:** 1 Misr University for Science and Technology, 6th of October, Egypt; 2 Medical Research Platform (MRP), Giza, Egypt; 3 School of Medicine, New Giza University (NGU), Giza, Egypt; 4 Faculty of Medicine, Tanta University, Tanta, Egypt; 5 Faculty of Medicine, University of Jordan, Amman, Jordan; 6 Faculty of Medicine, Mansoura University, Mansoura, Egypt; 7 Department of Medicine, College of Medicine and Health Sciences, An-Najah National University, Nablus, 44839 Palestine; 8 Faculty of Medicine, Alexandria University, Alexandria, Egypt; 9 Sheikh Khalifa Medical City, Abu Dhabi, UAE; 10 Faculty of Medicine, Al-Quds University, Jerusalem, Palestine; 11 Faculty of Pharmacy Clinical Department, Alexandria University, Alexandria, Egypt; 12 Faculty of Medicine, Suez Canal University, Ismailia, Egypt; 13 Emergency Medicine Department, Elsheikh Zayed Specialized Hospital, Elsheikh Zayed City, Egypt

**Keywords:** Health care, Information technology

## Abstract

Large language models (LLMs) like ChatGPT have potential applications in medical education such as helping students study for their licensing exams by discussing unclear questions with them. However, they require evaluation on these complex tasks. The purpose of this study was to evaluate how well publicly accessible LLMs performed on simulated UK medical board exam questions. 423 board-style questions from 9 UK exams (MRCS, MRCP, etc.) were answered by seven LLMs (ChatGPT-3.5, ChatGPT-4, Bard, Perplexity, Claude, Bing, Claude Instant). There were 406 multiple-choice, 13 true/false, and 4 "choose N" questions covering topics in surgery, pediatrics, and other disciplines. The accuracy of the output was graded. Statistics were used to analyze differences among LLMs. Leaked questions were excluded from the primary analysis. ChatGPT 4.0 scored (78.2%), Bing (67.2%), Claude (64.4%), and Claude Instant (62.9%). Perplexity scored the lowest (56.1%). Scores differed significantly between LLMs overall (*p* < 0.001) and in pairwise comparisons. All LLMs scored higher on multiple-choice vs true/false or “choose N” questions. LLMs demonstrated limitations in answering certain questions, indicating refinements needed before primary reliance in medical education. However, their expanding capabilities suggest a potential to improve training if thoughtfully implemented. Further research should explore specialty specific LLMs and optimal integration into medical curricula.

Artificial intelligence (AI) is a multidisciplinary field focused on developing machines and programs capable of replicating intelligent behavior^1^. Such machines are intended to perform tasks that require human intelligence. These tasks may vary depending on the industry; however, they usually revolve around the ability to learn, rationalize, and comprehend abstract concepts^1^.

AI was first established as a scientific discipline at the Dartmouth Summer Research Project in 1955^2^. However, over the past decade, the study of AI has experienced exponential growth and advances^3^. This was observed in different industries that integrated AI into their scope of work^3^. Despite the huge impact of these technologies in various industries, their application in medical field remains limited^3^.

In November 2022, a new AI model called ChatGPT was launched by OpenAI. ChatGPT, also known as Chat Generative Pre-trained Transformer, is a Large language model (LLM) with a trained parameter count of 175 billion^4^. This AI model gained significant attention because of its remarkable capability to carry out complex natural language tasks^4^. It was developed using deep learning algorithms, which are designed to learn and recognize patterns in data, to respond in a human-like manner to the user’s prompts^4^. Nevertheless, this technology is not exclusive to OpenAI, similar LLMs such as Bard, Google and Bing AI, and Microsoft have been recently launched to public use^5,6^.

The recent development and launch of multiple advanced LLMs has raised the question about their impact on medical education. Integration of advanced LLM may offer great opportunities to enhance the process of medical education such as improving teaching methodologies, personal studying, and the evaluation of one’s performance^7^. LLMs could play a huge role in curriculum development, personalized study plans and learning materials, and medical writing assistance^7^. Moreover, such advances may greatly benefit academics, especially when it comes to generating high-quality exams.^8^ However, blindly adopting such measures may present serious problems such as bias, misinformation, and overreliance—which may manifest as cheating in cases where exams are held online—that may hinder the development of medical students^7,9^. Recent studies have been conducted to assess the performance of AI chatbots on various board examinations. Lauren et al. explored the performance of ChatGPT on dermatology Specialty Certificate Examination (SCE) and found that ChatGPT-4 was capable of passing the exam with a score of 90.5%^10^. Furthermore, a similar study conducted on the United States Medical Licensing Examination (USMLE) found that ChatGPT was able to achieve a score similar to that of a third year medical student and provide a logical explanation for each answer^11^. Despite these impressive results, LLMs were still shown to perform poorly in questions that are ranked as high-order thinking questions^11,12^. This highlights the importance of further assessing the capabilities of such LLMs on different medical exam databases.

In this study, we evaluate the performance and clinical reasoning ability of various chatbots, including ChatGPT, on questions from the medical board examinations of the United Kingdom (such as MRCP, MRCS, RCOG, etc.). This article aims to assess the ability to use AI chatbots as a reliable medical educational tool for students undertaking medical board examinations.

## Methods

### Artificial intelligence

Various AI chatbots including ChatGPT 3.5, ChatGPT 4.0, Bard, Bing, Perplexity, Claude, and Claude-instant (accessed through Poe) have been used to generate natural linguistic responses to text inputs in a conversational manner. These AI modules are based on large databases that are used to train and lead to the generation of coherent and logical conversations that are appropriate to the context of the specified input.

### Input source and data abstraction

In this prospective study that was carried out from July 1st to July 31st, 2023, 440 multidisciplinary board-style test questions with public access from various sample questions provided by official sites of board exams were used to assess the performance of multiple artificial intelligence language modules with access to large datasets. Included sample questions were retrieved from board examination websites including MRCS, MRCP, RCPCH, RCOG, RCOopth, MRCPsych, FRCR (physics), FRCA, and MCEM in addition to sample obstetrics and gynecology questions provided by BMJ. Moreover, all inputs used were a true representation of real-exam scenarios assessing the performance of these AI models in a wide range of advanced medical disciplines. The inputs were further evaluated by being systematically assessed to ensure that none of the test answers, explanations, or exam-related content were recorded on the chatbots’ databases. Two researchers independently assessed each question for leakage by searching for both a sentence and a full question on Google and the C4 database^13^, which is included in most chatbots.^14^ The Google search was modified by the date filter “before:2022,1,1”—which represents the latest date accessible to the training of ChatGPT—and quotation marks for a sentence of the question and the full question. All questions that were leaked to Google, whether before or after 2022, or C4 database were excluded from the primary analysis. Furthermore, all sample test questions were screened to ensure the removal of questions containing visual or audiological inputs such as clinical images, graphs, and clinical audio inputs. After screening and excluding 17 questions containing images (all from pediatrics section), 423 board-style items involving multiple medical disciplines were advanced to data extraction and analysis. While using ChatGPT-4 and Bard, we made sure to not activate the web-search feature in these chatbots.

### Statistical analysis

The extracted data was then clustered into two categories, with the output = 1 representing that the AI module answered the question correctly, and an output = 0 representing a false or no answer. Subsequently, the data was analyzed using Cochran’s Q test and assessed for difference between chatbots with a significance level of p = 0.05. Further pairwise analysis was conducted using Bonferroni Correction with a significance level of p = 0.002. Statistical analysis was carried out using Jamovi^15^, and SPSS^16^. Whenever chatbots refused to answer on account of not giving medical advice, we considered this datum missing.

## Results

In this study, we assessed the performance of various AI modules in solving board-style questions including the MRCS, MRCP, RCPCH, RCOG, RCOopth, MRCPsych, FRCR (physics), FRCA, and MCEM. A total of 423 questions were included in the final analysis, the chatbot output was recorded and compared to the standardized question answer.

### Assessment of test set leakage

We found 7 questions leaked to the C4 database all of which are from the obstetrics and gynecology specialty and came from the MRCOG website. We found 18 MCQ questions leaked to Google while filtering by date, and an additional 51 leaked questions if filtering was off (present on Google after 1st January 2022). Of the 18 questions found on Google pre-2022, 12 were from the ophthalmology specialty, three from internal medicine, and three from pediatrics. All leaked questions from the mentioned sources totaled 97 questions. (Table 1).

**Table 1:** Frequencies of questions by leakage and specialty.

Leakage	Specialty	C4 leakage	Leakage in Google before 2022	Leakage in Google after 2022
Not leaked	Anaesthesia	12	12	9
	Emergency Medicine	10	10	10
	Internal Medicine	197	194	127
	Obstetrics and Gynecology	11	18	18
	Opthalmology	49	37	36
	Pediatrics	97	94	93
	Psychiatry	5	5	5
	Radiology	10	10	10
	Surgery	25	25	25
	GPT	0	0	0
Leaked	Anaesthesia	0	0	3
	Emergency Medicine	0	0	0
	Internal Medicine	0	3	70
	Obstetrics and Gynecology	7	0	0
	Opthalmology	0	12	13
	Pediatrics	0	3	4
	Psychiatry	0	0	0
	Radiology	0	0	0
	Surgery	0	0	0
	GPT	0	0	0

### Assessment results

Out of 333 questions that were not leaked, 310 questions were MCQ, 13 were true/false, and three were choose (n) from many. The highest number of questions that were not leaked were from the internal medicine Sect. (127), followed by pediatrics (93), ophthalmology (36), surgery (25), obstetrics and gynecology (11), emergency medicine (10), radiology physics (10), anaesthesia (9), and psychiatry (5) (Tables 1, 2).

**Table 2:** Frequencies of questions by type of question and leakage.

Leakage	Type of question	C4	Before 2022	After 2022
Not leaked	Choose (n) from Many	4	4	3
	MCQ	399	388	317
	True/False	13	13	13
Leaked	Choose (n) from Many	0	0	1
	MCQ	7	18	89
	True/False	0	0	0

**Figure 1:**
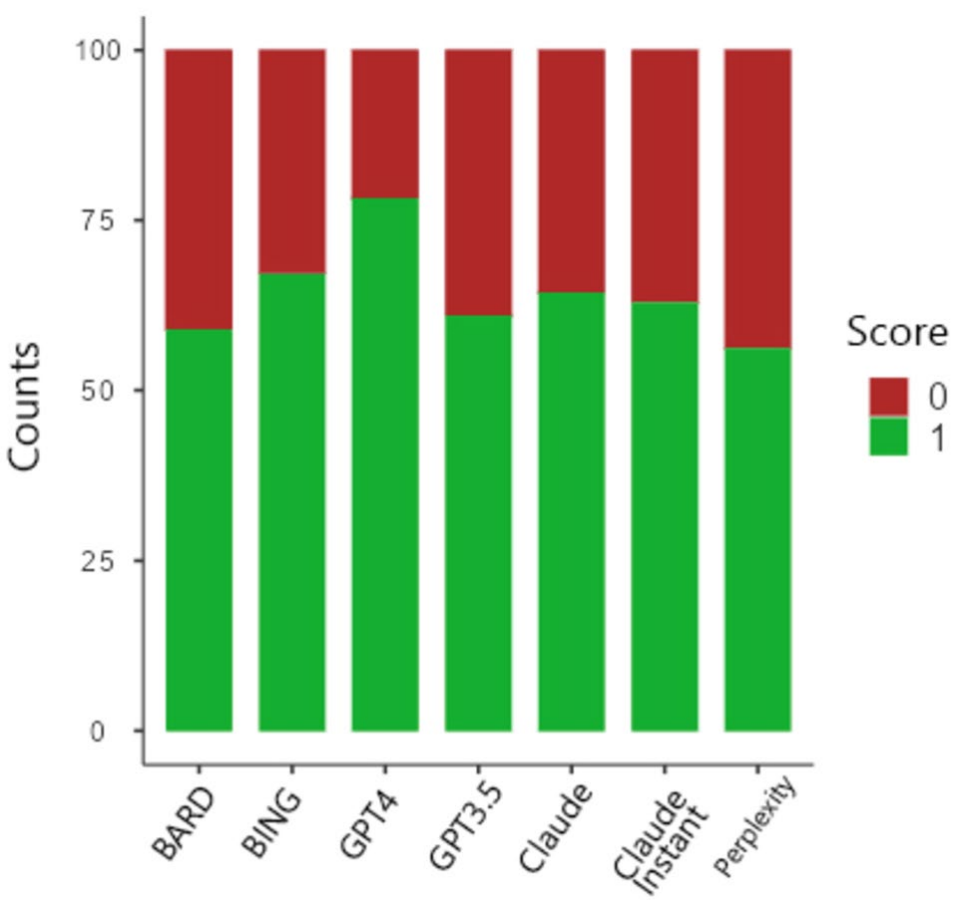
Frequencies of wrong (0) and correct (1) answers by chatbot.

On Average, ChatGPT 4.0 scored the highest with an average of 78.2% in answering questions that were neither leaked in Google before or after 2022, or in C4 database, followed by Bing (67.2%), Claude (64.4%), and Claude Instant (62.9%). On the other hand, Perplexity scored the lowest (56.1%). (Table 3; Fig. 1).

**Table 3:** Mean score (after removal of leaked questions) per chatbot.

ChatBot	Mean
Score	
Bard	0.589
Bing	0.672
ChatGPT 4 Paid	0.782
ChatGPT3.5	0.61
Claude	0.644
Claude Instant	0.629
Perplexity	0.561

All chatbots scored higher in MCQ questions (mean = 0.663) than choose (n) from many questions (mean = 0.381) while they scored lowest in true/false questions (mean = 0.187). All chatbots scored highest in emergency medicine (mean = 0.829), followed by psychiatry (mean = 0.771), anaesthesia (0.619), and internal medicine (0.677). However, they scored lowest in radiology physics questions (0.214) and surgery (0.543) (Tables 4, 5).

**Table 4:** Mean score by chatbot and question type.

	Type of question	Mean	SD
ChatGPT4	Choose (n) from Many	0.3333	0.577
	MCQ	0.8065	0.396
	True/False	0.3077	0.48
ChatGPT 3.5	Choose (n) from Many	0.3333	0.577
	MCQ	0.629	0.484
	True/False	0.2308	0.439
Bard	Choose (n) from Many	0.3333	0.577
	MCQ	0.6097	0.489
	True/False	0.1538	0.376
Perplexity	Choose (n) from Many	0.3333	0.577
	MCQ	0.5871	0.493
	True/False	0	0
Claude	Choose (n) from Many	0	0
	MCQ	0.6742	0.469
	True/False	0.0769	0.277
Bing	Choose (n) from Many	0.6667	0.577
	MCQ	0.6871	0.464
	True/False	0.3077	0.48
ClaudeInstant	Choose (n) from Many	0.6667	0.577
	MCQ	0.6452	0.479
	True/False	0.2308	0.439

**Table 5:** Mean score by chatbot and specialty.

	Specialty	N	Mean	SD
ChatGPT 3.5	Anaesthesia	9	0.778	0.441
	Emergency Medicine	10	0.9	0.316
	Internal Medicine	127	0.661	0.475
	Obstetrics and Gynecology	11	0.636	0.505
	Opthalmology	36	0.556	0.504
	Pediatrics	93	0.559	0.499
	Psychiatry	5	0.8	0.447
	Radiology	10	0.2	0.422
	Surgery	25	0.56	0.507
ChatGPT4	Anaesthesia	9	0.667	0.5
	Emergency Medicine	10	1	0
	Internal Medicine	127	0.858	0.35
	Obstetrics and Gynecology	11	0.636	0.505
	Opthalmology	36	0.778	0.422
	Pediatrics	93	0.763	0.427
	Psychiatry	5	1	0
	Radiology	10	0.4	0.516
	Surgery	25	0.6	0.5
Bard	Anaesthesia	9	0.778	0.441
	Emergency Medicine	10	0.8	0.422
	Internal Medicine	127	0.622	0.487
	Obstetrics and Gynecology	11	0.545	0.522
	Opthalmology	36	0.583	0.5
	Pediatrics	93	0.581	0.496
	Psychiatry	5	0.8	0.447
	Radiology	10	0.2	0.422
	Surgery	25	0.44	0.507
Perplexity	Anaesthesia	9	0.667	0.5
	Emergency Medicine	10	0.7	0.483
	Internal Medicine	127	0.606	0.491
	Obstetrics and Gynecology	11	0.182	0.405
	Opthalmology	36	0.556	0.504
	Pediatrics	93	0.591	0.494
	Psychiatry	5	0.6	0.548
	Radiology	10	0	0
	Surgery	25	0.52	0.51
Claude	Anaesthesia	9	0.444	0.527
	Emergency Medicine	10	0.8	0.422
	Internal Medicine	127	0.669	0.472
	Obstetrics and Gynecology	11	0.545	0.522
	Opthalmology	36	0.722	0.454
	Pediatrics	93	0.634	0.484
	Psychiatry	5	0.8	0.447
	Radiology	10	0.1	0.316
	Surgery	25	0.68	0.476
Bing	Anaesthesia	9	0.444	0.527
	Emergency Medicine	10	1	0
	Internal Medicine	127	0.646	0.48
	Obstetrics and Gynecology	11	0.455	0.522
	Opthalmology	36	0.639	0.487
	Pediatrics	93	0.817	0.389
	Psychiatry	5	0.8	0.447
	Radiology	10	0.4	0.516
	Surgery	25	0.44	0.507
ClaudeInstant	Anaesthesia	9	0.556	0.527
	Emergency Medicine	10	0.6	0.516
	Internal Medicine	127	0.677	0.469
	Obstetrics and Gynecology	11	0.636	0.505
	Opthalmology	36	0.583	0.5
	Pediatrics	93	0.656	0.478
	Psychiatry	5	0.6	0.548
	Radiology	10	0.2	0.422
	Surgery	25	0.56	0.507

A Cochran's Q test was conducted to assess whether there were differences in performance between the seven samples: Perplexity, GPT3.5, Bard, Claude Instant, Claude, Bing, and GPT4. The results of the Cochran's Q test were statistically significant, χ2(6) = 68.640238, *p* < 0.001, indicating significant differences in performance between the samples overall. (Table 6).

**Table 6:** Related-samples Cochran’s Q test summary.

Total N	326
Test statistic	68.640
Degree of freedom	6
Asymptotic sig.(2-sided test)	< 0.001

**Figure 2:**
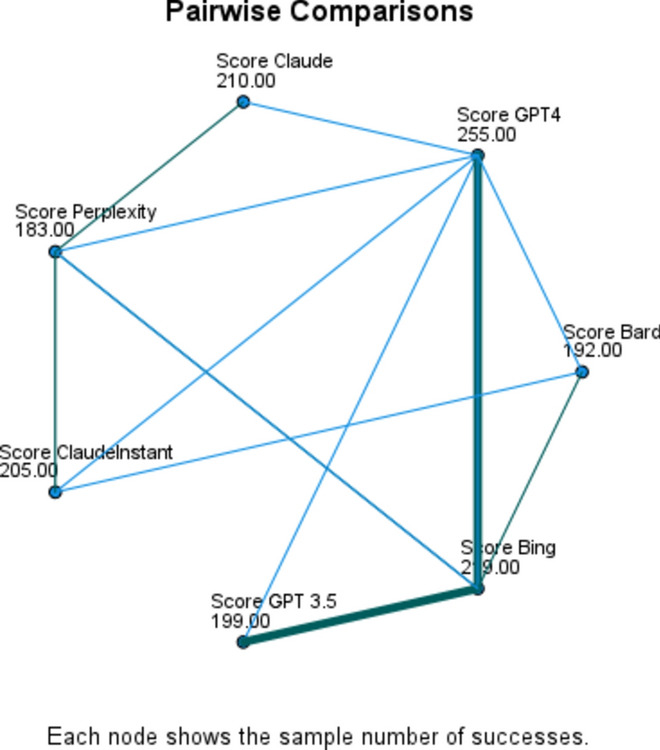
Pairwise comparison between chatbots.

**Figure 3:**
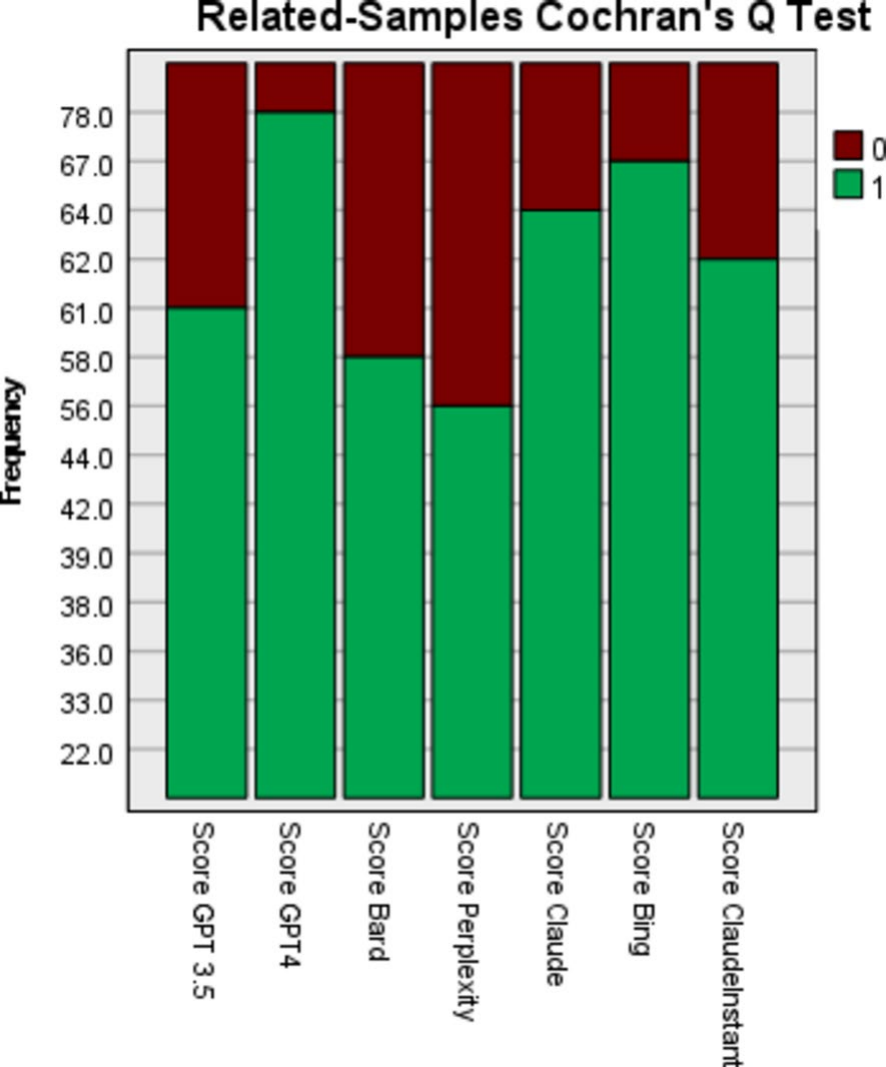
Related-samples Cochran Q Test.

Further pairwise comparisons were conducted with a Bonferroni correction to pinpoint where the differences existed between pairs of samples. Analysis revealed that ChatGPT4 significantly outperformed all other samples, scoring higher than Perplexity (*p* < 0.001), ChatGPT 3.5 (*p* < 0.001), Bard (*p* < 0.001), Claude Instant (*p* < 0.001), Claude (*p* < 0.001), and Bing (*p* < 0.001), suggesting that ChatGPT 4 was superior to all other models tested. Moreover, Perplexity scored significantly lower than several other models, it performed worse than ChatGPT4 (*p* < 0.001), and Bing (*p* < 0.001). A summary of pairwise comparisons are presented in Table 7 in addition to Figs. 2 and 3.

**Table 7:** Pairwise comparisons.

Sample 1-Sample 2	Test Statistic	Std. Error	Std. Test Statistic	Sig	Adj. Sig.^a^
Perplexity- Bard	0.028	0.030	0.918	0.359	1.000
Perplexity- GPT 3.5	0.049	0.030	1.632	0.103	1.000
Perplexity- ClaudeInstant	−0.067	0.030	−2.244	0.025	0.521
Perplexity- Claude	−0.083	0.030	−2.754	0.006	0.124
Perplexity- Bing	−0.110	0.030	−3.672	< 0.001	0.005
Perplexity- GPT4	0.221	0.030	7.345	< 0.001	< 0.001
Bard- GPT 3.5	0.021	0.030	0.714	0.475	1.000
Bard- ClaudeInstant	−0.040	0.030	−1.326	0.185	1.000
Bard- Claude	−0.055	0.030	−1.836	0.066	1.000
Bard- Bing	−0.083	0.030	−2.754	0.006	0.124
Bard- GPT4	0.193	0.030	6.427	< 0.001	< 0.001
GPT 3.5- ClaudeInstant	−0.018	0.030	−0.612	0.540	1.000
GPT 3.5- Claude	−0.034	0.030	−1.122	0.262	1.000
GPT 3.5- Bing	−0.061	0.030	−2.040	0.041	0.868
GPT 3.5- GPT4	−0.172	0.030	−5.713	< 0.001	< 0.001
ClaudeInstant- Claude	0.015	0.030	0.510	0.610	1.000
ClaudeInstant- Bing	0.043	0.030	1.428	0.153	1.000
ClaudeInstant- GPT4	0.153	0.030	5.101	< 0.001	< 0.001
Claude- Bing	−0.028	0.030	−0.918	0.359	1.000
Claude- GPT4	0.138	0.030	4.591	< 0.001	< 0.001
Bing- GPT4	0.110	0.030	3.672	< 0.001	0.005

Each row tests the null hypothesis that the Sample 1 and Sample 2 distributions are the same. Asymptotic significances (2-sided tests) are displayed. The significance level is .050.

^a^Significance values have been adjusted by the Bonferroni correction for multiple tests.

### Assessment results for leaked questions

All chatbots scored higher on questions leaked to the C4 database except for ClaudeInstant which performed worse on the seven questions leaked to the common crawl database (0.571 ± 0.535) than other questions (0.631 ± 0.483). Bard got all questions leaked to the C4 database correctly compared to a lower score of 0.585 ± 0.493 for other questions. Questions leaked to Google before the predetermined date of 1/1/2022, however, did not show any correlation with chatbot performance. In fact, all chatbots performed worse on these questions than on other questions. The breakdown of the results of the assessment of chatbots for leaked questions is presented in Table 8.

**Table 8:** Assessment of chatbot answer accuracy in answering leaked questions.

	C4 Leakage	N	Missing	Mean	SD
GPT3.5	0	414	1	0.618	0.486
	1	7	0	0.714	0.488
GPT4	0	415	0	0.786	0.411
	1	7	0	0.857	0.378
Bard	0	412	3	0.585	0.493
	1	7	0	1	0
Perplexity	0	415	0	0.571	0.496
	1	7	0	0.857	0.378
Claude	0	414	1	0.65	0.478
	1	7	0	0.714	0.488
Bing	0	415	0	0.67	0.471
	1	7	0	0.714	0.488
ClaudeInstant	0	415	0	0.631	0.483
	1	7	0	0.571	0.535

	Google leakage	N	Missing	Mean	SD
GPT3.5	0	404	1	0.619	0.486
	1	18	0	0.611	0.502
GPT4	0	405	0	0.79	0.408
	1	18	0	0.722	0.461
Bard	0	403	2	0.596	0.491
	1	17	1	0.529	0.514
Perplexity	0	405	0	0.58	0.494
	1	18	0	0.444	0.511
Claude	0	404	1	0.666	0.472
	1	18	0	0.333	0.485
Bing	0	405	0	0.672	0.47
	1	18	0	0.611	0.502
ClaudeInstant	0	405	0	0.64	0.481
	1	18	0	0.389	0.502

## Discussion

In this study, we examined the performance of various publicly available LLMs on questions derived from standardized United Kingdom medical board examinations. This was done to explore their potential use as educational and test preparation tools for medical students/doctors in the United Kingdom. The Seven AI models used in the study were ChatGPT-3.5, ChatGPT-4, Bard, Perplexity, Claude, Bing, and Claude Instant. Three formats of questions were given to the AI models: multiple choice, true/false, and “choose N from many” questions.

Our results showed statistically significant variations in the average scores for each AI model. We found that ChatGPT-4 had the best performance and overall scores. Meanwhile, Perplexity and Bard had the worst performance among the seven AI models. The remaining four AI models performed averagely, with no significant difference in performance between them. Despite ChatGPT-4 scoring the highest average across multiple-choice and true/false questions, it scored the lowest on “Choose N from many” questions (25%). In terms of average scores based on question format, the multiple-choice questions yielded the highest scores overall, with the different LLMs averaging between 60 and 81% correct (overall average 66%). In comparison, performance was lower for true/false and “Choose N from many” formats. The true/false questions proved to be the most challenging—LLMs scored between 0 and 31% correct, with Perplexity unable to answer any question correctly. On the “Choose N from many” questions, performance was better than true/false, but worse than multiple choices. LLMs averaged 25–50% correct, with Claude Instant and Bing scoring 50%, the highest of any model in this format. These results highlight the differences in how well LLMs can handle various question types. Even an LLM that scores highly on one format, like GPT-4 on multiple choice, does not necessarily perform as well on other formats like “Choose N from many.” This suggests that the models have strengths and weaknesses based on their prompt structure. Overall, their ability to reason through and answer medical exam questions accurately across different formats remains limited compared to that of human experts. However, performance is steadily improving, underscoring the importance of continued research on refining LLM skills for complex tasks.

Similar to our study, many other papers have shown the remarkable ability of LLMs to pass reputable exams. Antaki et al. demonstrated the ability of ChatGPT to pass ophthalmology examinations at the level of a first-year resident^17^. Furthermore, it was found to pass the United States Medical Licensing exam with a score equal to that of an average third-year medical student^11^. However, most of these studies were limited to OpenAI’s ChatGPT alone. In contrast, our study explored seven LLMs including ChatGPT. This allowed for a more comprehensive performance analysis of currently available LLMs. Moreover, this is the first study to explore the performance of LLMs in various United Kingdom medical board examinations. Our findings can be summarized into three major themes:^17^ ChatGPT-4 remains the best average performer among AI models (2). The performance of AI models may differ depending on the formulation of the prompt question (3). The use of AI models as a secondary educational tool is propitious; however, using such models as a primary source is not recommended before further refining.

Recent advancements in LLMs, specifically ChatGPT, seem to disrupt current medical education and assessment models. Trends in AI improvement indicate that the implementation of this technology in all fields, including medicine, is inevitable. The notion of continuous improvement in these models can be seen by the documented increase in ChatGPT performance on the Medical Licensing Exam of the United States of 60% when compared with previous studies that found a much lower accuracy rate on comparable tests^11,18^. Additionally, in our study, ChatGPT-4 scored 78% correct overall which is 18% higher than the previously reported score on the USMLE examinations. Considering that these exams are intended to test medical personnel at a similar level, it would be reasonable to assume that this may indicate the continuous improvement of such models. Therefore, such models must be treated as opportunities to improve all aspects of medical education in an ethical and responsible manner. Efforts must be directed at exploring further methods to enhance the ability of LLMs to answer prompts with higher accuracy. Currently, the performance of LLMs suggests that their use as an educational tool must be as an adjuvant source in a comprehensive educational approach rather than as the primary source^19^. This takes into consideration the current limitations of such LLMs in scientific and mathematical knowledge and applications^19^.

An important aspect to consider with the rise of these models is the ethical concern of potential misuse of malicious intent, such as cheating. The risk of such misuse should be weighed against the expected gains from this technology. Therefore, educational institutions must work to counteract the misuse and prevent the unethical exploitation of this technology. If implemented correctly, this technology may lead to substantial improvements in medical education. Further studies must be conducted to continuously monitor this improvement and explore other ways to improve medical education through these advanced LLMs.

### Factors affecting chatbot accuracy

The varying accuracy of chatbot answers can be attributed to the low sample size of questions we were able to acquire. For instance, all chatbots performed worse on the 13 true/false (0.187 ± 0.392) and in four choose (n) from many (0.286 ± 0.46) questions than 406 MCQs (0.664 ± 0.473). This unbalanced sample may hinder the generalizability of our results in questions other than MCQs. As for the leaked questions on Google, the websites that hosted them varied, as some were locked behind a paywall, such as on Scribd website^20^, others were in a PDF format, as a part of questions samples^20,21^, while others were on flashcards on websites such as Quizlet.^22^ Investigating leakage of exam questions to databases included in publicly available LLMs can be very advantageous for academic or research purposes. It can be done, akin to our approach, by search the C4 database or by implementing guided prompting to answer medical questions from a specific dataset.^23^

### Prompting

As mentioned previously, the disparity between the percentage of correct answers in MCQ questions and true/false questions can be explained by multiple factors. The first factor is prompting. Prompt engineering refer to the practice of carefully designing and optimizing the prompts or instruction given to AI systems (such as ChatGPT) to improve their performance on specific tasks. This can help communicate user intent and desired outputs to LLMs. It also improves performance, provides customizable interaction, allow incorporation of external knowledge, control output features, and mitigate biases. The published research on prompt engineering for medical users is scarce. However, many preprints^24–27^ suggested some practices for good prompt engineering. Firstly, it is advised to provide clear specific instructions as ambiguous prompts can lead to unclear or irrelevant responses. Moreover, users are encouraged to continuously test and tweak prompts based on model responses to improve responses.^24–27^

### Specialization of chatbosts

While all chatbots included in this study can be described as LLMs which provide text generation based on user-developed prompt, it is better to deal with available options as specialized tools for different tasks. While more research is needed with future development of medically oriented LLMs, we can deduce from each chatbot descriptions and characteristics the different uses in which each chatbot may excel its peers. For instance, from included chatbots, only Perplexity and Bing AI provide sources, with Perplexity being able to refine sources more-accurately to academic ones. Moreover, Perplexity has a GPT-4 co-pilot which may enhance results of answering medical questions, but we did not assess it. On the other hand, only ChatGPT 4.0 (paid version) and Claude has file analysis features which make them able to summarize texts and analyze sheets and codes. Claude, ChatGPT (both free and paid versions) are not currently available in some regions which may encumber users (both researchers, medical practitioners, and medical students) from numerous countries from accessing them. It is interesting to see how the current AI-revolution folds out and what new tools can contribute to medical education and medical decision making.

### Leakage

Data leakage significantly impacts the accuracy of chatbots, particularly in the domain of medical question answering. Data leakage occurs when the training data of a model inadvertently includes information from the test set, leading to an overestimation of the model's true performance. Brookshire et al.^28^ explored this effect by studying the effect of data leakage on the neural networks’ ability to correctly identify a range of disorders using EEG. In this example, the leakage of EEG segments to the training set and its reappearance in the test set leads to inflated model accuracy. Leakage can create a false sense of reliability and even an inflated accuracy^29^. A model trained on leaked data may appear to perform exceptionally well during testing, but this performance does not translate to real-world scenarios where the model must answer previously unseen questions. This discrepancy is particularly concerning for medical students who rely on the chatbot for studying and acquiring accurate medical knowledge. Misleading performance metrics can lead to overconfidence in the chatbot's responses, potentially spreading incorrect or incomplete medical information.

### Limitations

This study had several limitations. First, due to financial limitations, we were limited to the sample questions provided free of cost on each respective board examination website. This resulted in a lower number of question prompts used than originally intended. Second, LLMs available to the public are continuously trained with new data over time. This may affect the applicability of these findings to the updated versions of each LLM. Furthermore, All chatbots displayed limitations when it came to true–false and "choose N" questions which may be explained by the small sample of these questions included in our study. The high number of MCQ-type questions may also lead to LLM performance inflation. However, despite these limitations, our study provides comprehensive analysis and insights into the strengths and limitations of the seven LLMs as an educational tool for the preparation of United Kingdom medical board examinations.

## Conclusions

This study offers fresh perspectives on how various publicly accessible AI chatbots performed when faced with UK medical board exam questions. The accuracy of the chatbots varied significantly, with ChatGPT-4 doing the best overall. According to our research, these AI models could be beneficial for medical students as secondary learning resources, but they still need to be improved before they can be used as main teaching tools. Prompt engineering and developing specialized medical LLMs could help improve performance. Overall, as LLMs develop, they provide promising chances to change medical education.

### Supplementary Information

Supplementary Information 1.

Supplementary Information 2.

Supplementary Information 3.

## Data Availability

The data used or generated during this study are presented in this publication. They can be found in the accompanying supplementary information files.
